# Thinking of norms—or being told what they are: The effect of social information on donation decisions

**DOI:** 10.1371/journal.pone.0321547

**Published:** 2025-04-25

**Authors:** Hagit Sabato

**Affiliations:** The Seymour Fox School of Education, The Hebrew University of Jerusalem, Mount Scopus, Israel; Queen Mary University of London School of Business and Management, UNITED KINGDOM OF GREAT BRITAIN AND NORTHERN IRELAND

## Abstract

The effect of social information (descriptive versus injunctive norms) on people’s donation decisions was examined in two studies. In Study 1 (N=376) participants received information about the norm (a high versus a low anchor) for each norm type, while in Study 2 (N=392) participants were instructed to think of the social norm (*what one ought to do* vs. *what most people do*) before their donation decision. Results suggest that when actual information was given (Study 1), a high anchor reduced participants’ initial willingness to donate—but among those who did decide to donate, the high anchor resulted in greater donation amounts than the low anchor. This pattern held true for both injunctive and descriptive norms. Merely thinking about the injunctive norm—without any anchor (Study 2)—increased donations, compared with thinking about the descriptive norm, or control conditions. Possible explanations, and the implications for charitable giving are discussed.

## Introduction

Enhancing people’s prosociality has been the subject of considerable attention in the decision-making literature, resulting in the recognition of various factors that affect one’s decision to help others [or not] (such as perceived benefits and costs [1]; the donors’ subjective well-being [2]; the number of potential givers or receivers [3]; information about the recipient’s characteristics [4]). One major factor that has been found to influence such decisions is social information, that is presented before the decision to guide people’s behavior (e.g., [5]).

Specifically, the common social norm—which represents the social expectation of an appropriate behavior—is a significantly effective source of information that people may rely on when deciding whether to help [6,7]. Social norms come in various types. The most commonly examined of these in past research are *injunctive* and *descriptive* norms: While the former refer to people’s beliefs or perceptions about what is morally correct (i.e., what ought to be done), the latter indicates people’s beliefs about what is actually done by most people in a given situation [6,8].

Although these two norms represent distinct theoretical concepts, they may be partially correlated (e.g., [9])—suggesting that people’s notions of what “ought to be done” may stem from what they know about the common behavior of others, while their assumptions about the common behavior may be a result of their perceptions of obligated behaviors [10]. There is solid evidence, however, for situations in which the influences of the two norms do not overlap—for example, when people endorse certain behaviors, but do not apply them in practice [6,11].

Both descriptive and injunctive norms are known to have a significant effect on behavioral decisions in various life domains (such as college drinking [12], energy saving [13], water use [14]), including prosocial inclinations (such as cooperation [15], tipping behavior [16]). However, studies examining the relative impact of various social norms on behavior have yielded mixed results [17,18,19,20]. The two norms are specifically relevant when considering prosocial actions, which often involve uncertainty and ambiguity as to what should be done, thus increasing the probability that people seek for and rely on social-norm information in their decisions [21,22].

### The role of social norms in prosocial decisions

In the context of prosociality, overall, research findings bear out the positive effect of descriptive norms on donations [21,23–26]. For example, Hysenbelli et al., [27] found that participants who were presented with high average donations by other people donated greater amounts (compared with participants in low anchor and control conditions). Agerström et al. [28] observed a similar positive effect of the descriptive norm on charitable giving, with a stronger impact when the norm was local (same university) rather than global (regional students). The influence of descriptive norms on prosocial decision-making may also be inferred from studies of people’s decision to avoid such information—a phenomenon known as *deliberate ignorance* [29]. In a recent study, Andersson et al., [30] presented participants with the option to reveal (or not) the proportion of participants who had previously donated to a charitable organization, before deciding whether to do so themselves. They found a positive effect of the norm on donations, such that participants who revealed the norm and learned that the majority had donated, were more likely to donate (than those who learned that only a minority had donated). Interestingly, participants who chose not to know the norm donated less frequently; but when they did donate, they contributed a greater amount, on average. This pattern suggests that different motivations are involved in people’s decision to learn about the descriptive norm (or not)—such as maintaining a positive self-image or valuing independence—leading variably to selfish and to prosocial decisions. Supporting this idea, previous research on the mechanisms behind the positive effect of descriptive norms on prosociality highlights various, and sometimes conflicting, mechanisms (e.g., social pressure [31], perceived impact and sense of personal involvement [32]).

It is important to note, however, that this positive effect of the descriptive norm on prosociality failed to replicate in several studies [33,34,35]—and in some studies even reversed, i.e., decreased donations [36]. Conversely, there has been evidence that injunctive norms also have a positive effect on prosociality [37,38,39]. For example, in Capraro and Rand’s study [40], participants exhibited greater prosocial behavior in economic games after being presented with injunctive cues.

Studies that directly compared the effect of injunctive versus descriptive norms on actual prosocial behavior have yielded mixed findings. A handful of studies have compared the effect of injunctive and descriptive norms on participants’ allocation decisions, using the Dictator Game (DG) method. Bicchieri and Xiao [41] examined the effect of fair versus selfish norms on allocation decisions, and found that descriptive norms are a stronger predictor of fair allocation decisions than injunctive norms, when the two norm types are in conflict. Using a similar Dictator Game method, Raihani and McAuliffe’s study [42] revealed different findings. Here, the norms included fair versus low anchors, and were presented either as descriptive (what “most participants did”) or as injunctive (what “is suggested to do”)—with the addition of control groups with the same numerical anchors. They found that the injunctive norms were associated with a greater likelihood of giving at least the target amount to one’s partner (compared with the other conditions). Interestingly, in that study, descriptive norms did not appear to affect the participants’ allocations decisions.

These contradictory findings—along with the mixed results in studies of the relative impact of norms in other behavioral contexts (as previously noted)—may stem from the different manipulations that were used for norm salience (e.g., providing a signal for social approval [43]; presenting a directive message of what constitutes “proper behavior” [44]; offering the advice of a reputed expert in the field [17]). In Bicchieri and Xiao’s study [41], for example, which looked at participants’ fair (vs. selfish) allocations, the anchors were “stingy” (20%) versus more generous (40%), and the injunctive norm was examined by perceptions of the behavior to be approved by others. Conversely, in Raihani and McAuliffe’s study [42], the anchors included fair allocation, and the injunctive norm was framed as the recommended course of action. The latter may be perceived as more distinct from the behavior of others (i.e., descriptive norm), and representing the common moral rule in a clearer fashion, thus having a greater impact on behavior [40]. This is also in line with Krupka and Weber’s study [45], in which participants were asked to choose between fair and selfish allocation options in a binary DG. In their experiment, an injunctive-norm focus (i.e., asking participants to think about others’ perceptions of what should be done) and a descriptive-norm one (i.e., asking participants to think about what others have done)—increased fair allocation decisions, in relation to the selfish option, to a similar degree as presenting them with the actual descriptive norm. Interestingly, participants’ allocations were affected both by focusing and by informational manipulations—even when they expected or observed low levels of prosociality. This finding may suggest that merely drawing attention to the social norm per se has a positive effect on allocation decisions.

These studies used the DG as the primary paradigm for studying the comparison between the impact of norms on prosociality, and yielded mixed findings. It is important to note, however, that when allocation behavior is factored in, the norm of equality appears to be the norm in common (e.g., [46])—as reflected in the norm that was adopted as anchor in most of these studies and in the DV’s that referred to fair vs, selfish allocations. However, in real-life contexts, donation behavior usually has no clear social rules or acceptable behavior, so is more ambiguous.

In line with previous research that found a greater effect of social information in ambiguous or less familiar situations (e.g., [21]), the present study sought to focus on donation decisions, in which no clear reference is available (be it perceived, or applied by others). In such ambiguous situations, people tend to seek social information, in order to compare themselves with others (e.g., considering their resources relative to those of others, in donation decisions—[47], or considering downward social information of others’ donation in comparison with one’s previous donation decision—[48]), and to rely on their own beliefs or assumptions about the behavior of others. Such assumptions, however, may be biased, as people often fail to correctly recognize the social norm, or misinterpret it.

### Misinterpreting social norms: perceptual biases in self- other comparisons

One systematic bias in norm estimation is evident in research on *pluralistic ignorance* [49,50]—a psychological state where a person believes their private attitudes and judgments are different from those of others, even though their outward actions are the same. This bias often stems from the mistaken belief that although others are acting similarly, they are experiencing different feelings—an assumption that may influence one’s decision-making. For example, in the context of prosocial decision making, pluralistic ignorance is one explanation for the *bystander effect* [51]. In this situation, although individuals bystanders’ may internally recognize an emergency and the need for help, they mistakenly assume that their fellow bystanders (who are not taking action) have concluded that intervention is unnecessary [52].

Another perceptional bias in self and others’ comparisons, is known as the *better than average* (BTA) effect. This effect suggests that people often view themselves as superior to others in various positive qualities, such as traits and behaviors [53,54]. This perception is illusory because, at the group level and assuming a normal distribution of the trait in question, most individuals cannot be above average [55,56]. In the context of the present study, research has shown that people perceive themselves to be more prosocial than average in terms of sharing, donation giving, and cooperation [57].

The present study aimed to examine the role of injunctive and descriptive norms in actual donations, both when social information is explicitly provided and when it is absent, and individuals need to rely on subjective estimations, instead. This aligns with the distinction made by Epley and Gilovich [58] between internal (self-generated) anchors and external ones (provided by an experimenter or other outside source). This highlights the importance of the anchor’s source in understanding its effect: when social norms are absent, individuals may rely on internal anchors, such as their personal estimations, which are more prone to egocentric biases or naïve beliefs—which in turn affect their decisions.

### The present research

To the best of my knowledge, as previously noted, most past research on the behavioral effect of injunctive and descriptive norms provided participants with actual information about what “ought to be done” or is socially approved (i.e., injunctive norms), or about most people’s behavior (i.e., descriptive norms). Studies that directly compared the relative effects of these two norms have yielded conflicting findings. Moreover, many of these studies involved experimental economic games focused on the equality norm, and are not always replicable in real-world situations [59,60,61]. In the present research, I sought first to examine the effect of specific given information of each type of norm, in actual donation decisions within a real-world context (Study 1). Next, I aimed to explore the effect of merely inducing people to think about the injunctive or descriptive norm on d

onation decisions, without providing them actual information about it (Study 2). The innovation of that method is that it allows participants to use their subjective perceptions of the norm (however naïve or biased these may be) to guide their own behavior. This systematic examination was expected to allow for a better understanding of the effect of social influences, on donation decisions, with and without actual information —a relatively ambiguous context in terms of the accepted behavior.

In the two studies, I first included a dichotomous question on the participant’s initial willingness to donate, followed by a question about the donation amount (for those who decided to donate). This allowed for the norm’s effect on each decision to be examined, as suggested by the *two-stage processing model of donation decisions* [62]. According to this model, when asked to donate, people first decide whether to provide any help at all, and then decide how much. Each such step involves a different mechanism behind the decision.

For exploratory reasons, in both studies I examined participants’ post-decisions emotions. This has the potential to shed light on the effect of norm types—not only on participants’ behavioral decisions, but on their emotional reactions following such decisions, as well. Emotions, as a primary psychological mechanism behind prosocial decisions [63], are also known to play a crucial role in facilitating future subsequent decisions [64]. Thus, exploring the emotional reaction after the donation decision may contribute to a broader understanding of the effects of norms and anchors.

I hypothesized that: (H1) When presented with specific information about the norm—namely, a given anchor (Study 1)—the provided information will affect the participants’ donation behavior. Given the mixed findings in the literature regarding the differential effects of descriptive and injunctive norms, I adopt a bidirectional approach: while norm type may shape participants’ responses in distinct ways, it is also possible that the anchoring effect will exert a stronger influence, overriding potential differences between norm types. This prediction is in line with previous research on the anchoring effect, that found that when participants are provided with external anchors (e.g., by the experimenter), they tend to focus more on those anchors, and are less influenced by other social cues [58]. It is also supported by findings from previous studies on norm-type effects, that found a tendency among participants to not fully distinguish between types of norms when no contradicting information is presented [41]. (H2) In the absence of a concrete anchor (Study 2), participants’ estimations would serve as inner anchors. Since such estimations are more likely to be biased, and are sensitive to social cues [58], I predicted that the injunctive norm would induce participants to donate more than a descriptive norm (a general assessment of others’ behavior) or the control conditions. This prediction is based on the nature of the injunctive content (what “ought to be done” that highlights one’s own moral values (e.g., [40]), and is prone to self-serving bias [57]. At the same time, vague thoughts about the donations of others may lead to relatively lower estimates, due to the *BTA effect* [57], thus justifying lower amounts.

To examine these hypotheses, I conducted two studies. The first sought to examine people’s donation decisions after being presented with an injunctive norm (i.e., the recommended behavior) as opposed to a descriptive one (i.e., what others would do), and with a control condition. In each condition, participants were exposed to a high versus low anchor. Study 2 used the same type of norms, without providing the social-norm information before the donation decision. Instead, participants were presented with a general instruction to think about the injunctive norm versus the descriptive one, and a control condition under which no instruction was given.

As previously noted, what “ought to be done” may be defined either as one’s perceptions of the behavior that would be approved by others (significant others, [65], or anonymous others, [41]) or as a more general “guiding rule” regarding what should be done ([42,66], also framed as “a personal norm” [67]). In both studies I adopted the latter, to avoid a possible conflation between people’s beliefs about others people’s perceived notions and their actual behavior (a mix that was found in previous studies—e.g., [10]).

To support this definition, I ran a pilot study that directly compared the effect of the two options of the injunctive norm’s manipulation described above on participants’ estimations of the norm, their donation decision, and their recommendations for others.

Data collection for the studies was carried out between April 2021 and July 2024 (4/25–6/11/21; 2/11–2/13/22; 5/7–7/1/24). The research was approved by the author’s department Ethics Committee. All participants were adults and signed a written consent at the start of the online questionnaire, prior to their inclusion in the studies. No attention or manipulation checks were used in the studies, so all participants were included in the analyses. However, data quality was ensured by reviewing all responses for outliers (e.g., participants who selected the same response for all items) and for the completeness of the questionnaires. All responses were valid and complete, and no exclusions were made from the dataset.

Data is available at https://osf.io/54t6m/?view_only=ab525fd2d1d04ea5ad4d0ce22d1f1d54

## Pilot study

One hundred and fifty-two participants (63.8% women, MAge = 25.30; SD = 3.23) completed a short online questionnaire. After being presented with a short introduction that explained that our lab had entered into a collaboration with a charity organization (see the full details in the main studies), participants were randomly assigned to one of two experimental conditions: (1) *Injunctive Norm–Self*, which highlighted the participant’s perception of the “right thing to do”: “*In case of winning the NIS 100 prize in the raffle, in your opinion is it recommended or not to donate to the charity?*” If they answered “Yes,” they were asked to write the recommended donation amount, in their opinion, between NIS 1–100, and (2) *Injunctive Norm—Others*, which highlighted others’ perceptions of the “right thing to do”: “*In case of winning the NIS 100 prize in the raffle, in your opinion do people around you would think that it is recommended or not to donate to the charity*?” Here too, if they answered “Yes,” they were asked to state the amount others would think is recommended to donate, between NIS 1–100. Next, they were asked for a donation (between NIS 1–100) and how much they would recommend others to donate, as described in the main studies.

### Results

The amount that participants were willing to donate ranged from *0* (34 participants) to *100* (30 participants)—*M* = 40.93, *SD* = 35.36.

Results of an independent sample’s t-test revealed a non-significant difference between the experimental conditions in participants’ answers to the manipulation question—i.e., the estimated amount (*t* = –.360, *p* = 0.473; Injunctive Norm–Self: *M* = 44.20, *SD* = 36.91; Injunctive Norm–Others: *M* = 46.43, *SD* = 39.05)—and in their willingness to donate (*t* =.327, *p* =.616; Injunctive Norm–Self: *M* = 41.88, *SD* = 34.63; Injunctive Norm–Others: *M* = 40.00, *SD* = 36.26) or in their recommendations for others (*t* = –.580, *p* =.905; Injunctive Norm–Self: *M* = 42.96, *SD* = 29.82; Injunctive Norm–Others: *M* = 45.92, *SD* = 31.77).

These non-significant differences between the two manipulations of injunctive norm are in line with previous findings of a negligible effect between a manipulated injunctive norm based on other people’s perceptions and a manipulated personal norm based on one’s perception of the recommended behavior, on one’s behavior (e.g., [68]). Since previous research pointed to a possible conflation between people’s beliefs about other people’s notions of what “ought to be done” and their actual behavior [10], in both studies I adopted the definition of the injunctive norm as a general “guiding rule” as to what should be done—rather than one’s perceptions of the socially approved behavior.

## Study 1

### Method

#### Participants.

Three hundred and seventy-six undergraduate university students (60.6% women, MAge = 25.46; SD=4.24) took part in the study, after being recruited through WhatsApp or social media (i.e., student groups). They were asked to complete a short online questionnaire, in return for entry into a raffle for a prize of NIS 100 (approximately US $33). Sample size was determined by a power analysis (α =.05) with G*Power software [69], that indicated that a sample of 382 participants would detect a small-to-medium effect size (*f*^*2*^ = 0.17), in a 2x3 ANOVA (Norm Type: Descriptive/Injunctive/Control Anchor: High/Low) between-subjects groups, α=0.05, power of 0.85.

#### Procedure.

The questionnaire began with an explanation that our lab is collaborating with a nonprofit charity organization that offers a broad spectrum of services to ill and disabled people (such as supportive health, and equipment lending services), to help them return to a normal life. Next, participants were randomly assigned to one of six experimental conditions in a 3 (**Norm Type**: Descriptive/ Injunctive/ Control) X 2 (**Ancho**r: High vs. Low) design. I included two control conditions, to examine the possibility of a simple anchoring effect on the donation amount due to the mere presence of the high vs. low number [42]. The text that participants were presented with in each condition is presented in Table 1.

**Table 1:** Experimental conditions’ descriptions.

Norm Type/ Anchor	Injunctive	Descriptive	Control
**High**	It is recommended to donate about NIS 70 (~17$).	Most of the students who participated in this study so far donated about NIS 70 (~$17).	Did you know? The average student uses enough ink to fill about 70 pens during their degree.
**Low**	It is recommended to donate about NIS 20 (~5$).	Most of the students who participated in this study so far donated about NIS 20 (~$5).	Did you know? The average student uses enough ink to fill about 20 pens during their degree.

Next, all participants were asked whether they would be willing to donate any part of the NIS 100 prize (a dichotomous question) if they were to win it in the raffle at the end of the experiment—and if so, how much (between NIS 1 to NIS 100). They were also told that they would be held to that decision if they win. All the money raised in this study was indeed subsequently donated to a nonprofit organization that provides the services described above. After their donation decision, participants rated their emotions about their decision (*pride, guilt, sadness, satisfaction, anger, joy, disappointment, happiness*). These emotions were presented in randomized order, and participants rated each of them on a 1–7 scale (from 1=*Not at all* to 7= *Very much*). All participants were then asked a few additional questions about their decision, that are discussed in the S1 File, and for demographic details.

### Results

#### Donation decision: willingness to donate.

To examine whether Anchor and Norm Type influenced the participants’ initial decision to donate or not (a dichotomous variable), as suggested by the two-stages model for donation decisions [62], I first conducted a hierarchic logistic regression analysis on the Willingness to Donate. Anchor (coded as Low = 0 and High = 1) and Norm Type (Dummy 1: Control = 0; Injunctive and Descriptive = 1; Dummy 2: Injunctive = 0; Control and Descriptive = 1) were included in the first step, and the two-way interactions in the second step.

The results revealed a significant effect of Anchor on Willingness to Donate, Wald (1) = 10.73, *B*= -.77, *p* =.001—such that participants were significantly more willing to donate in the Low-Anchor condition.

The interaction between Anchor and Dummy 1 (i.e., the comparison between the control condition and the two Norm Type conditions) was significant, Wald (1) = 5.02, *B* =−1.32, *p* =.025, such that the effect of the Anchor was significant under the two Norm Type conditions, Wald (1) = 15.59, *B* = -1.17, *p* <.001; while in the control condition, there was no significant difference between the two anchors—, Wald (1) = 0.00, *p* = 1.00—ruling out a possible effect of the numerical anchor itself. The number and percentage of donors in each condition and for each anchor are presented in Table 2.

**Table 2:** Number and percentage of donors in each condition and for each anchor.

Willingness to Donate/Norm Type	Yes	No
**Injunctive** ^*^		
** Low Anchor**	54 (81.8%)	12 (18.2%)
** High Anchor**	36 (62.1%)	22 (37.9%)
**Descriptive** ^*^		
** Low Anchor**	48 (82.8%)	10 (17.2%)
** High Anchor**	37 (56.1%)	29 (43.9%)
**Control**		
** Low Anchor**	51 (75%)	17 (25%)
** High Anchor**	45 (75%)	15 (25%)

* p<0.05 ** p<.01 *** p<.001

Because the results revealed a significant effect on participants’ willingness to donate under the various conditions, the following analysis of the donation amounts included only donors.

#### Donation decision: donation amount.

**Fig 1:**
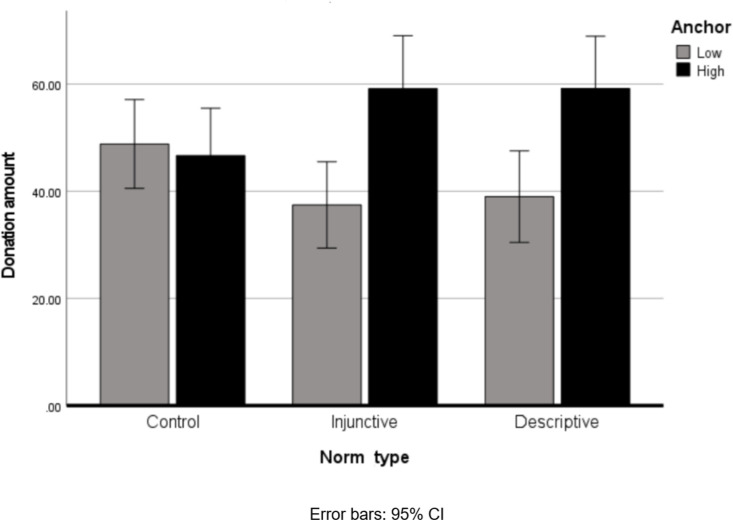
Donation amount as a function of Norm Type and Anchor.

A two-way ANOVA was conducted on donation amounts, with Norm Type (Descriptive, Injunctive, control) and Anchor (High vs. Low) as predictors. Results revealed a significant effect for Anchor: *F*(1, 265) = 12.82, *p* <.001, *η*_*p*_^*2*^ =.046—such that the High Anchor induced higher donation amounts (*M* = 54.41, *SD* = 28.15) than the Low Anchor (*M* = 41.73, *SD* = 31.98). More importantly for the purposes of the current research, the interaction between Norm Type and Anchor was significant: *F*(2, 265) = 4.52, *p* =.012*, η*_*p*_^*2*^ =.033, as presented in Fig 1. A simple-effects analysis revealed that the effect of the Anchor was significant both in the Injunctive Norm, *F*(1, 265) = 11.23, *p* <.001, *η*_*p*_^*2*^ =.041, and in the Descriptive one, *F*(1, 265) = 9.40, *p* =.002, *η*_*p*_^*2*^ =.034—such that under a High Anchor participants donated more (Injunctive norm: *M* = 59.16 *SD* = 21.66; Descriptive Norm: *M* = 59.19, *SD* = 30.60) than under a Low one (Injunctive norm: *M* = 37.46 *SD* = 29.31; Descriptive Norm: M= 39.00, SD= 30.52)—but not in the control condition *F*(1, 265) = 0.123, *p* =.726, *η*_*p*_^*2*^ =.000. Notably, the effect of the anchor was very similar in both the Injunctive and the Descriptive Norm conditions.

Next, I sought to conduct an exploratory examination of the possible effect of the norm types and anchors on the participants’ post-decisions emotions ratings.

#### Post-decision emotions.

The four positive emotions (*pride, satisfaction, joy, happiness*) were highly correlated (α =.91), and a Warm Glow Index was computed as their average. The four negative emotions (*guilt, sadness, anger, disappointment*) were also highly correlated (α =.83), and their average was computed as a Cold Prickle Index (based on [70]).

A three-way ANOVA of Warm Glow, with Norm Type, Anchor and Willingness to Donate as predictors, revealed only a significant main effect for Willingness to Donate, *F*(1, 364) = 92.24, *p* < 0.001, *η*_*p*_^*2*^ =.202)—such that participants who decided to donate reported more intense positive emotions *(M* = 4.33, *SD* = 1.72) than those who decided not to (*M* = 2.60, *SD* = 1.63).

**Fig 2:**
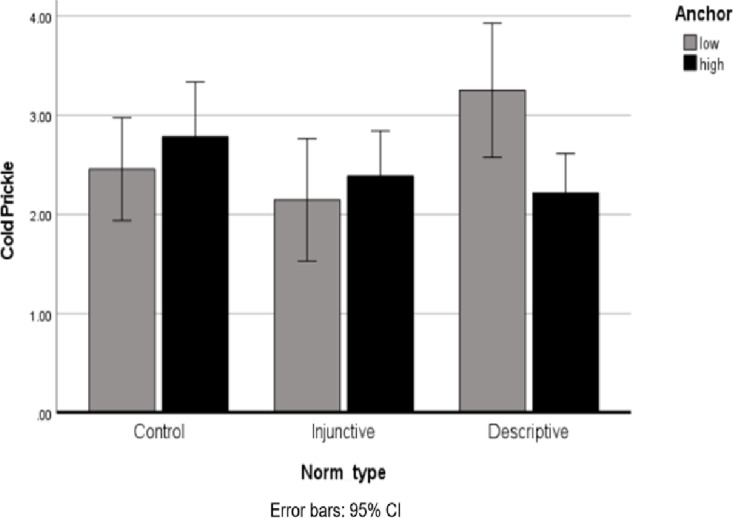
Cold Prickle (negative emotions) among non-donors, as a function of Norm Type and Anchor.

A three-way ANOVA of Cold Prickle, with Norm Type, Anchor and Willingness to Donate as predictors, revealed a significant main effect for Willingness to Donate, *F* (1, 364) = 33.70, *p* < 0.001, *η*_*p*_^*2*^ =.085)—such that participants who decided to not to donate reported more acute negative emotions (*M* = 2.46, *SD* = 1.29) than those who decided to donate (*M* = 1.77, *SD* = 1.01). In addition, the interaction between Norm Type, Anchor and Willingness to Donate was significant, *F* (2, 364) = 3.56, *p* =.029, *η*_*p*_^*2*^ =.019. To better understand this interaction, I ran a two-way ANOVA of Cold Prickle, with Willingness to Donate and Anchor as predictors, separately for each condition. The interaction between Anchor and Willingness to Donate was significant only in the Descriptive Norm condition, *F* (1, 120) = 7.84, *p* =.006, *η*_*p*_^*2*^ =.061, and not in the control condition: *F* (1, 124) =.766, *p* =.38, *η*_*p*_^*2*^ =.006, or in the Injunctive Norm condition, *F* (1, 120) =.012, *p* =.915, *η*_*p*_^*2*^ =.000. A further simple-effect analysis in this condition revealed that participants who decided not to donate and had been presented with a Low Anchor, reported significantly more acute negative emotions (*M* = 3.25, *SD* = 1.76) than those who decided not to donate and had been presented with a High Anchor (*M* = 2.2, *SD* = 1.25), *F* (1, 120) = 7.26, *p* =.008, *η*_*p*_^*2*^ =.057, see Fig 2. This difference was not significant after the decision to donate*, F* (1, 120) =.900, *p* =.345, *η*_*p*_^*2*^ =.007.

This interesting finding may suggest that although the two norms yielded a similar pattern in Willingness to Donate and in donation amounts, they had a different effect on participant’s post-decision emotions—specifically following the decision *not to* donate, as I discuss further below.

The results of Study 1 highlighted an interesting effect of anchoring in the context of social norms—namely, that a high anchor induces a lesser willingness to donate than a low anchor—in both the Descriptive and the Injunctive Norm conditions. However, when the analysis is limited to those who chose to donate, social information that cited higher donated amounts increased participants’ donations, both in the Injunctive and in the Descriptive Norm conditions. Notably, the effect of the anchor was very similar in both conditions.

While in Study 1 participants were presented with specific information (high vs. low anchor) in each norm type, Study 2 sought to examine donations to the same cause, when the social-norm information is not explicitly provided. Instead, participants were guided simply to think of the norm (injunctive or descriptive) with no anchor being present. This allowed me to explore the effect of the various norms when actual social information is not presented.

## Study 2

### Method

#### Participants.

Three hundred and ninety-two undergraduate university students (56.1% women, MAge = 24.90; SD=3.53) were recruited through the database of prospective studies of the university. They were asked to complete a short online questionnaire, in return for an entry into a raffle for a prize of NIS 100 (Approximately $33). The sample size was determined as in Study 1, by power analysis (*α* =.05) with G*Power software [69], that indicated that a sample of 381 participants would detect a small-to-medium effect size (*f* =.17), in a one-way ANOVA, with three groups (experimental conditions: Descriptive Norm, Injunctive Norm, and Control), power = 0.85.

#### Procedure.

In the first screen, participants were told that our lab is working with a charity organization (as in Study 1). Participants were then randomly assigned to one of three experimental conditions: **(1) Descriptive Norm**, in which they were asked about their estimations for the common behavior in the donation decision (*“In case of winning the NIS 100 prize in the raffle, in your opinion would most students taking part in the study donate or not to the charity?”,* If they answered “Yes,” they were asked to write the amount they thought most students would donate, between NIS 1–100). **(2) Injunctive Norm**, in which they were asked what ought to be done in such donation decision in their opinion *(“In case of winning the NIS 100 prize in the raffle, in your opinion is it recommended or not to donate to the charity?”* If they answered “Yes,” they were asked to write the recommended donation amount, in their opinion, between NIS 1–100**) (3) Control**, in which no preliminary question was presented. It is important to note, due to differences in phrasing between Hebrew (the spoken language of the study) and the English translation presented here, that the Descriptive Norm refers to the participant’s perception of the behavior of others, whereas the Injunctive Norm refers to the participant’s perception of what is considered appropriate or desirable behavior (i.e., “the right thing to do”).

Next, as in Study 1, participants were presented with a donation request (their willingness to donate in case of winning, and if so, how much); rated their post-decision’s emotions; and answered a few exploratory questions that are discussed in the S1 File.

### Results

#### Donation decision: willingness to donate.

The amount that participants were willing to donate ranged from *0* (108 participants) to *100* (68 participants)—*M* = 38.04, *SD* = 35.51.

I first conducted a logistic regression analysis on the Willingness to Donate (a dichotomous variable) as a function of Norm Type (coded as Control = 0; Descriptive = 1, Injunctive = 2)—as suggested by the two-stage model for donation decisions [62]—to examine whether the Norm Type influenced the participants’ initial decision to donate, or not. The results revealed no significant difference between the three groups in terms of the participants’ willingness to donate: Wald (2) = 3.09, *p* =.213 (comparison between Injunctive and Control, *p* =.261; comparison between Descriptive and Control: *p* =.503). Because there was no significant difference in participants’ willingness to donate as a function of the experimental condition, all participants were included in further analyses, with the non-donors’ donation coded as zero. However, the same analyses with donors only revealed a similar significant effect (see S1 File). The number and percentage of donors in each condition are presented in Table 3.

**Table 3:** Number and percentage of donors in each condition.

Willingness to Donate/Norm Type	Yes	No
**Injunctive**	98 (77.8%)	28 (22.2%)
**Descriptive**	87 (68%)	41 (32%)
**Control**	99 (71.1%)	39 (28.3%)

#### Donation decision: donation amount.

**Fig 3:**
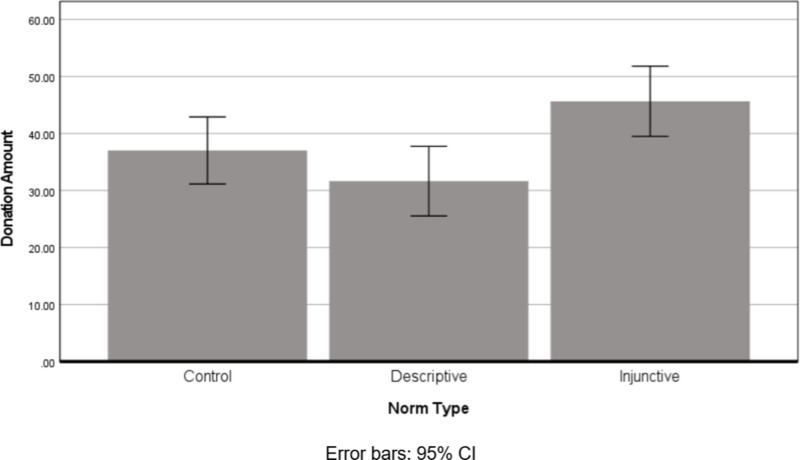
Donation amount as a function of Norm Type.

A one-way ANOVA of donation amounts, with Norm Type (descriptive, injunctive, control) as a factor, was conducted. Results revealed a significant effect for Norm Type, *F* (2, 389) = 5.13, *p* =.006, *η*_*p*_^*2*^ =.026, see Fig 3. A post-hoc analysis further suggested that, as predicted, participants in the Injunctive Norm condition donated significantly more (*M* = 45.65, *SD* = 38.79) than those in the Descriptive Norm one (*M* = 31.64, *SD* = 32.19), *p* = 0.002, and more than participants in the control group (*M* = 37.02, *SD* = 34.24), *p* = 0.047; while the two latter conditions did not significantly differ (*p* =.213).

#### A mediation model: estimated amount.

To gain a better understanding of the effect of each norm on decisions, I conducted an exploratory examination of the participants’ answers to the initial questions of the manipulation (the amount they believe that one should donate, and the amount they believe most students would donate—hereafter, the *Estimated Amount*). Results of a T-test analysis of the Estimated Amount revealed a significant effect: *t* (252) = −8.98, *p* < 0.001—such that participants in the Injunctive Norm condition suggested significantly higher donation amounts (*M* = 47.70, *SD* = 42.25) than those in the Descriptive Norm condition (*M* = 9.76, *SD* = 22.07). Note that the same analysis while excluding estimated zero donations (78.1% in the Descriptive Norm condition vs. 29.4% in the Injunctive Norm condition), also yielded a significant, albeit smaller, difference (Injunctive norm: M = 68.29, SD = 33.80; Descriptive norm: M = 44.60, SD = 26.06; *t* (114) = −3.39, p <.001).

Next, I conducted a mediation analysis, using the SPSS PROCESS macro model 4, with bootstrap techniques and 5,000 resamples [71], with Norm Type as a predictor (x); Estimated Amount as mediator (m); and Donation as the DV (y). The Norm Type in the model included only the two conditions in which the estimated amount was measured during the experimental manipulation, and not the Control condition, that did not include a manipulation.

As previously noted, Norm Type significantly predicted Estimated Amount (*t* = 8.98, *p* <.001, 95% CI [29.62, 46.25]); and Estimated Amount significantly predicted Donation (*t* = 14.39, *p* <.001, 95% CI [.614,.809]), and significan*t*ly mediated the effect of Norm Type on donation (B = 27.019, SE = 4.009, 95% CI [19.72, 35.38]). However, the direct effect of Norm Type on Donation remained significant (*t* = -3.41, *p* <.001, 95% CI [−20.51, −5.50]). This suggests *t*hat the Estimated Amount partially explains the effect of Norm Type on Donation—such that the anchor that participants had in mind was significantly different in the two conditions, and influenced their donation decision.

#### Post-decision emotions.

The four positive emotions were highly correlated (α =.92), and a Warm Glow Index was computed as their average. The four negative emotions were also highly correlated (α =.84), and their average was computed as a *Cold Prickle Index* (based on [70]).

A two-way ANOVA of Warm Glow with Norm Type and Willingness to Donate as predictors, revealed a significant main effect for Willingness to Donate: *F*(1, 386) = 153.55, *p* < 0.001, *η*_*p*_^*2*^ =.285)—such that participants who decided to donate reported more intense positive emotions (*M* = 4.46, *SD* = 1.58) than those who decided not to (*M* = 2.27, *SD* = 1.48). The effect of Norm Type was not significant: *F*(2, 386) = 1.54, *p* =.215, *η*_*p*_^*2*^ =.008)—nor was the interaction between Norm Type and the Willingness to Donate, *F*(2, 386) = 3.05, p =.70, *η*_*p*_^*2*^ =.004).

A two-way ANOVA of Cold Prickle, with Norm Type and Willingness to Donate as predictors, revealed a significant main effect for Willingness to Donate, *F*(1, 386) = 47.60, *p* < 0.001, *η*_*p*_^*2*^ =.110)—such that participants who decided to not to donate reported more acute negative emotions (*M* = 2.41 *SD* = 1.23) than those who decided to donate (*M* = 1.60, *SD* =.93). The effect of Norm Type was not significant, *F* (2, 386) = 2.93, *p* =.243, *η*_*p*_^*2*^ =.007). The interaction between Norm Type and Willingness to Donate was marginally significant, *F*(2, 386) = 2.93, *p* =.055, *η*_*p*_^*2*^ =.015). A simple-effect analysis revealed that the decision to donate did not result in a significant difference in the participants’ negative emotions, *F* (2, 386) =.26, *p* =.77, *η*_*p*_^*2*^ =.001. Conversely, the decision not to donate tended to spark more acute negative emotions in the control condition (*M* = 2.7, *SD* = 1.18) than in the norm conditions (Injunctive: *M* = 2.25, *SD* = 1.28; Descriptive: *M* = 2.22, *SD* = 1.22), *F* (2, 386) = 2.94, *p* =.054, *η*_*p*_^*2*^ =.015. This pattern is at odds with the results of Study 1, that found more acute negative emotions for participants who decided not to donate and had been presented with a high-anchor descriptive norm. However, since in Study 2 participants were asked to think of the norm, and the mean estimated amount in the Descriptive norm was low (as presented earlier), the decision not to donate did not confront them with a high standard, so did not negatively affect their emotions. Another possible explanation may be the nature of the priming manipulation, which involved a more deliberate way of thinking. Deliberate processes cause people to rely more on their cognition than on their emotional responses [72,73], and may therefore somewhat suppress negative feelings after the decision not to help.

## General discussion

In two studies, I examined participants’ donation behavior when presented with injunctive vs. descriptive cues. Study 1 presented participants with an injunctive norm (i.e., what is “ought to be done”) versus a descriptive one (i.e., what “most people do”)—while providing a high versus low anchor of donation amounts. Study 2 repeated the experiment with the same norm types, using a priming manipulation that merely directed participants to think about what “ought to be done” as opposed to “what most people do”—without providing any specific information or anchors.

The results revealed an initial decrease in participants’ willingness to donate when presented with the high anchor, compared with the low one. However, among those who did decide to donate, the high anchor did increase the amounts donated (Study 1). Interestingly, these patterns were very similar for both injunctive and descriptive norms—suggesting that the anchor has a greater effect on donation behavior than the norm type (injunctive or descriptive). When no specific information was given (Study 2), a positive effect was found for the injunctive cue over the descriptive one--—that is, participants donated significantly more under a priming manipulation that directed them merely to think about the injunctive norm (what they perceive to be the right thing to do), than about the descriptive norm (what they believe most people do), and in the control condition.

These findings highlight the importance of effective use of social information in inducing people to donate more in real-life situations (rather than allocation preferences in economic games, which are not always replicable in real-life decisions [59,60,61]). They add to the existing literature of direct comparisons between descriptive and injunctive norms, while focusing on real-life, actual behavior, where the common social norm may be somewhat vague (as opposed, for example, to the equity norm in allocation decisions—e.g., [42]).

The very similar effect found for descriptive and injunctive norms (compared with the control condition) on donation amounts when low and high anchors were provided, may indicate that people tend to rely on the provided anchor, and pay less attention to its framing (descriptive or injunctive) when the social norm is unclear. This finding is in line with the distinction made by Epley and Gilovich [58] between external anchors that are provided by an external source, and tend to be less affected by social cues (compared with self-generated anchors). It is in line with Bicchieri and Xiao’s [41] findings of a positive effect for both descriptive and injunctive content on allocation behavior, when there is no conflict between the two norms—suggesting that, in the absence of a direct comparison, people do not fully distinguish between the two types of norms.

However, when post-decision emotions are examined, an interesting pattern was found: when being presented with a low Descriptive Norm, participants reported more acute negative emotions after their decision not to donate at all. This may indicate the power of social comparison, which is more apparent with a descriptive norm than with an injunctive one. Such a comparison appears to trigger a negative emotional reaction after a behavioral decision that is not in line with even a low common standard. Future research is needed to explore this effect and its possible ramifications (e.g., for subsequent decisions).

Interestingly, in the current study the anchoring effect [58,74] revealed different pattern in each step of the donation decision (i.e., the initial decision to donate, and the decision on donation amount—a distinction suggested by the two-step model of donation decisions [62]), such that the high anchor decreased participants’ initial willingness to donate, while it enhanced donations among those who decide to donate. This pattern suggests that a high anchor has both motivating and demotivating effects on donation behavior. The initial negative impact of a high anchor on people’s willingness to donate in both types of norms (compared with the control and a low anchor) is consistent with past research that found that people are less inclined to help when presented with a high donation request [75], with a message that asks for a “generous contribution” [76], or with a high tip suggestion [77]. This suggests that some people may regard the high anchor as unrealistic, unfair, or simply irrelevant—and therefore legitimately ignored, or possibly even justification for forgoing any donation whatsoever.

In terms of research on the effectiveness of nudges on behavior, the high anchor may be perceived as an illegitimate manipulative attempt to shape one’s behavior [78,79], as it offends people’s basic desire for perceived control and freedom of choice in their decision-making [80,81,82]—thereby negating or even countering the nudge’s purpose. However, this negative effect may be dependent on situational and personal factors. For example, the prospective recipient of help in the present study was a charitable organization—a general cause that is known to spark less empathic arousal than a single, identified victim [4]. A reaction to a high anchor in such a case may be less engaging. Indeed, a previous study that presented participants with a single identified individual as the prospective recipient [27] found no effect of the anchor on the willingness to help (*Yes* or *No*), and a positive effect of the high anchor on donation amounts. It is possible that people are more willing to accept the higher anchor in the case of a single identified victim, given their greater emotional response in such instances [4]. The direct comparison between these two situations (identified individuals vs. general cause) in the context of the anchoring effect is a topic for further research.

From a different perspective, the more complex pattern of the high anchor effect echoes the previously noted finding by Endersson et al. [30] that participants’ decision to avoid the descriptive norm resulted both in less frequent donations and greater mean-donation amounts. Their call for further examination of individual differences in participants’ motivations or characteristics that may have interacted with the experimental manipulation (prompting both prosocial and selfish donation decisions) holds true in the present research as well. Thus, aside from situational factors that may attenuate the effect of high anchors on behavior, future research is needed to explore specific characteristics of donors and non-donors—such as lower social desirability, social conformity, and self-presentation considerations, to identify potential moderators of the anchoring effect.

When no specific information (i.e., no anchors) is provided and people freely consider the norm (Study 2), the injunctive cue results in significantly greater donations than the descriptive one. An injunctive norm is linked to the perception of what one *should do* (i.e., “the right decision”) in a given situation, directing people to invoke their noble values in their decision [83]. Such consideration is likely to create a high anchor for the donation to charity. Conversely, thinking about what *most people do* (descriptive norm) may create a lower anchor for donation due to possible bias in Self-Other comparisons. Previous studies of *pluralistic ignorance* [49, 50] found that people tend to mistakenly believe that their private attitudes, beliefs or behaviors are different from the group norm—even when others’ observed behavior belies this assumption. This misperception affects decision-making processes, as individuals adapt their behavior to their biased perception of others [49,50]. Specifically, in the context of prosocial behavior it has been found that people underestimate others’ prosocial traits and behavior, due to the *BTA effect* [57]. This idea is supported by the exploratory mediation analysis, which found that inner estimations indeed partially mediated the association between norm type and donations.

Another possible explanation for the effect of priming people to think about the injunctive norm on their subsequent behavior may be related to the greater wish for coherence between one’s values and one’s behavior under this prime. This proposition is in line with Schwartz’s *norm activation model* for altruism [84], which suggests that when social norms are translated into personal norms, the general values become internalized and associated with one’s self-image, and are therefore more likely to enhance one’s moral and altruistic behavior. Studies of the role of personal norms in various prosocial behaviors have found support for this model (e.g., recycling behavior [85]; cooperation in the face of social dilemma [86]; consumption behavior [87]; subsequent sharing decisions [83]).

The findings are also consistent with research on the effect of conscious thinking on behavior [88], and specifically on the positive effect of introspection on donation decisions. For example, Pittarello and Kogut’s study [89] found that asking for participants’ opinion about a given charity campaign, as opposed to merely asking for a donation, increased donations. Another recent study [72] found that merely guiding people to think about their donation decision before making it, reduces their biases in their donation decision (i.e., makes their behavior more aligned with their intentions). Similarly, in the previously cited study by Krupka and Weber [45], guiding participants to focus on the social norm (i.e., what others perceive as the right choice) increased their prosocial choices. The current study findings suggest that such a *focusing effect* may yield different patterns when the guidance highlights people’s inner beliefs and perceptions of the norm, rather than their perceptions of others’ behavior.

The results call for caution in the use of social information nudges to enhance donations in either injunctive or descriptive norms. First, the more complex effect of the high anchor on donations obtained in Study 1 highlights the need to carefully consider its use, given the negative effect it may have on those who choose to refrain from donating altogether, when encountering it. Moreover, the results of Study 2 suggest an alternative and more useful social information nudge may be to encourage people to think about the recommended behavior without providing them with a specific anchor. This method allows them to create their own desirable reference which—as was found—tends to be relatively high, while preserving their sense of control over their donation decision. Thus, beyond its empirical and theoretical contribution, the current research has important implications for fundraising, suggesting effective ways of using social information to enhance donations.

### Study limitations

The current research has several limitations. First, it was conducted among students, and the descriptive norm manipulation referred to students’ behavior. Although this is a common method in social norms research [7], it is important that future research will include other populations to allow for a generalization of the findings.

Second, the injunctive norm in this study referred to the general guiding rule (what “ought to be done”), rather than to others’ perception of it. This decision was supported by the results of the pilot study, and in line with previous findings [68] of no significant difference between an injunctive norm manipulation based on other people’s perceptions and one’s perception of the recommended behavior, on one’s behavior. However, since the pilot study examined this potential difference in the context of free thinking, with no particular anchor, future research might examine the role of Self-Others cues also when specific information, i.e., anchors, is provided.

Finally, the estimated amounts were not measured in the control condition, so the comparison of the amount participants assumed included the norm-type conditions only. Future research might include a similar question in control conditions, as well (at the end of the experiment), to allow a comparison with natural situations, too, where no initial instruction was given.

## Supporting information

S1 File Supplementary Materials .
(DOCX)

## References

[1] Pittarello A, Caserotti M, Rubaltelli E. “Three is better than two”: increasing donations with the attraction effect. Br J Psychol. 2020;111(4):805–22. doi: 10.1111/bjop.12428 31617591

[2] Sabato H, Bar-Ilan S. Pleasure or meaning: Subjective well-being orientations and the willingness to help close versus distant others. J Happiness Stud. 2023;24(6):2013–37.

[3] Chiang YS, Hsu YF. The asymmetry of altruistic giving when givers outnumber recipients and vice versa: a dictator game experiment and a behavioral economics model. J Econ Psychol. 2019;73:152–60.

[4] Kogut T, Ritov I. The “Identified Victim Effect”: an identified group, or just a single individual?. J Behav Decis Making. 2005;18:157–67.

[5] Coffman L, Featherstone C, Kessler J. Can social information affect what job you choose and keep?. Am Econ J Appl Econ. 2017;9(1):96–117.

[6] Cialdini RB, Reno RR, Kallgren CA. A focus theory of normative conduct: recycling the concept of norms to reduce littering in public places. J. Pers. Soc. Psychol 1990;58(6):1015–26.

[7] Van Teunenbroek C, Bekkers R, Beersma B. Look to others before you leap: a systematic literature review of social information effects on donation amounts. Nonprofit Volunt Sect Q. 2020; 49(1): 53–73.

[8] Kallgren C, Reno R, Cialdini R. A focus theory of normative conduct: when norms do and do not affect behavior. Pers Soc Psychol Bull. 2000;26(9):1002–12.

[9] Brauer M, Chaurand N. Descriptive norms, prescriptive norms, and social control: an intercultural comparison of people’s reactions to uncivil behaviors. Eur J Soc Psychol. 2010;40(3):490–9.

[10] Eriksson K, Strimling P, Coultas J. Bidirectional associations between descriptive and injunctive norms. Organ Behav Hum Decis Process. 2015;129:59–69.

[11] Lapinski MK, Rimal RN. An explication of social norms. Commun Theory. 2005;15(2):127–47. doi: 10.1111/j.1468-2885.2005.tb00329.x

[12] Borsari B, Carey KB. Descriptive and injunctive norms in college drinking: a meta-analytic integration. J Stud Alcohol. 2003;64(3):331–41. doi: 10.15288/jsa.2003.64.331 12817821 PMC2431131

[13] Allcott H. Social norms and energy conservation. J Public Econ. 2011;95(9–10):1082–95.

[14] Bhanot S. Rank and response: a field experiment on peer information and water use behavior. J Econ Psychol. 2017;62:155–72.

[15] Kandul S, Lanz B. Public good provision, in-group cooperation and out-group descriptive norms: a lab experiment. J Econ Psychol. 2021;85:102382.

[16] Lynn M. The effects of injunctive and descriptive tipping norms on tipping behavior and motives. J Behav Exp Econ. 2021;95:101786. doi: 10.1016/j.jbee.2021.101786

[17] Bailey J, Nofsinger J, O’Neill M. Retirement plan contribution decision factors: the role of social norms. J Bus Manag. 2004;9(4):327–44.

[18] Bohner G, Schlüter LE. A room with a viewpoint revisited: descriptive norms and hotel guests’ towel reuse behavior. PLoS One. 2014;9(8):e104086. doi: 10.1371/journal.pone.0104086 25084348 PMC4118982

[19] Goldstein N, Cialdini R, Griskevicius V. A room with a viewpoint: using social norms to motivate environmental conservation in hotels. J Consum. Res. 2008;35(3):472–82.

[20] Reese G, Loew K, Steffgen G. A towel less: social norms enhance pro-environmental behavior in hotels. J Soc Psychol. 2014;154(2):97–100. doi: 10.1080/00224545.2013.855623 24765814

[21] Hoover H. Nudges as norms: evidence from the NYC taxi cab industry. J Econ. Psychol. 2022;92:102535.

[22] Wittek P, Liu YH, Darányi S, Gedeon T, Lim IS. Risk and ambiguity in information seeking: eye gaze patterns reveal contextual behavior in dealing with uncertainty. Front Psychol. 2016;7:1790. doi: 10.3389/fpsyg.2016.01790 27909418 PMC5112274

[23] Frey B, Meier S. Social comparisons and pro-social behavior: testing ‘conditional cooperation’ in a field experiment. Am Econ Rev. 2004;94:1717–22.

[24] Goeschl T, Kettner S, Lohse J, Schwieren C. From social information to social norms: Evidence from two experiments on donation behaviour. Games. 2018;9(4):91.

[25] Sasaki S. Majority size and conformity behavior in charitable giving: Field evidence from a donation-based crowdfunding platform in Japan. J Econ Psychol. 2019;70:36–51.

[26] Croson R, Handy F, Shang J. Keeping up with the Joneses: the relationship of perceived descriptive social norms, social information, and charitable giving. Nonprofit Mgmnt Ldrshp. 2009;19(4):467–89. doi: 10.1002/nml.232

[27] Hysenbelli D, Rubaltelli E, Rumiati R. Others’ opinions count, but not all of them: Anchoring to ingroup versus outgroup members’ behavior in charitable giving. J Judgment Decis Making. 2013;8(6):678–90.

[28] Agerström J, Carlsson R, Nicklasson L, Guntell L. Using descriptive social norms to increase charitable giving: the power of local norms. J Econ Psychol. 2016;52:147–53.

[29] Vu L, Soraperra I, Leib M, van der Weele J, Shalvi S. Ignorance by choice: A meta-analytic review of the underlying motives of willful ignorance and its consequences. Psychol Bull. 2023;149(9–10):611–35. doi: 10.1037/bul0000398 38713751

[30] Andersson PA, Erlandsson A, Västfjäll D. Norm avoiders: the effect of optional descriptive norms on charitable donations. J Behav Decis Making. 2022;35(1):e2244.

[31] Lay S, Zagefka H, González R, Álvarez B, Valdenegro D. Don’t forget the group! The importance of social norms and empathy for shaping donation behaviour. Int J Psychol. 2020;55(4):518–31. doi: 10.1002/ijop.12626 31608442

[32] Gugenishvili I, Francu R, Koporcic N. I give a dime if you do, too! The influence of descriptive norms on perceived impact, personal involvement, and monetary donation intentions. J Consum Behav. 2022;21(2):167–79.

[33] Kubo T, Shoji Y, Tsuge T, Kuriyama K. Voluntary contributions to hiking trail maintenance: Evidence from a field experiment in a national park, japan. Ecological Economics. 2018;144:124–8. doi: 10.1016/j.ecolecon.2017.07.032

[34] Murphy J, Batmunkh N, Nilsson B, Ray S. The impact of social information on the voluntary provision of public goods: a replication study. Res Exp Econ. 2015;18:41–50.

[35] Bergquist M, Nyström L, Nilsson A. Feeling or following? A field‐experiment comparing social norms‐based and emotions‐based motives encouraging pro-environmental donations. J Consum Behav. 2020;19(4):351–8.

[36] Meyer A, Yang G. How much versus who: which social norms information is more effective?. Applied Economics. 2016;48(5):389–401.

[37] Gugenishvili I, Colliander J. I will only help if others tell me to do so! The simultaneous influence of injunctive and descriptive norms on donations. Volunt Sect Rev. 2023;14(3):421–44. doi: 10.1332/204080521x16442337687557

[38] Lu T, Liang D, Hong M. Shaping future generosity: the role of injunctive social norms in intertemporal pro-social giving. J Econ Psychol. n.d.;102:102717.

[39] Smith JR, McSweeney A. Charitable giving: the effectiveness of a revised theory of planned behaviour model in predicting donating intentions and behaviour. Community Appl Soc Psy. 2007;17(5):363–86. doi: 10.1002/casp.906

[40] Capraro V, Rand D. Do the right thing: experimental evidence that preferences for moral behavior, rather than equity or efficiency per se, drive human prosocial. J Judgment Decis Making. 2018;13(1):99–111.

[41] Bicchieri C, Xiao E. Do the right thing: but only if others do so. J Behav Decis Making. 2009;22(2):191–208.

[42] Raihani NJ, McAuliffe K. Dictator game giving: the importance of descriptive versus injunctive norms. PLoS One. 2014;9(12):e113826. doi: 10.1371/journal.pone.0113826 25493945 PMC4262257

[43] Reno RR, Cialdini RB, Kallgren CA. The transsituational influence of social norms. J Pers Soc Psychol. 1993;64(1):104–12.

[44] Schultz W, Khazian A, Zaleski A. Using normative social influence to promote conservation among hotel guests. Social Influence. n.d.;3(1):4–23.

[45] Krupka E, Weber R. The focusing and informational effects of norms on pro-social behavior. J Econ Psychol. 2009;30(3):307–20.

[46] Güth W, Schmittberger R, Schwarze B. An experimental analysis of ultimatum bargaining. J Econ Behav Organ. n.d.;3(4):367–88.

[47] Pittarello A, Motsenok M, Dickert S, Ritov I. When the poor give more than the rich: The role of resource evaluability on relative giving. J Behav Decis Mak. 2023;36(1):e2293.

[48] Croson R, Shang J. The impact of downward social information on contribution decisions. Exp Econ. 2008;11(2):221–33.

[49] Miller D, McFarland C. When social comparison goes awry: the case of pluralistic ignorance. Soc Compar Contemp Theory Res. n.d.:287–313.

[50] Prentice DA, Miller DT. Pluralistic ignorance and alcohol use on campus: some consequences of misperceiving the social norm. J Pers Soc Psychol. 1993;64(2):243–56. doi: 10.1037//0022-3514.64.2.243 8433272

[51] Latané B, Darley J. The unresponsive bystander: why doesn’t he help?. 1970.

[52] Miller D. A century of pluralistic ignorance: what we have learned about its origins, forms, and consequences. Front Soc Psychol. 2023;1(1):1260896. doi: 10.1234/example.doi

[53] Alicke M. Global self-evaluation as determined by the desirability and controllability of trait adjectives. J Pers Soc Psychol. 1985;49:1621–30.

[54] Allison S, Messick D, Goethals G. On being better but not smarter than others: the muhammad ali effect. Social Cognition. 1989;7:275–96.

[55] Taylor SE, Brown JD. Illusion and well-being: a social psychological perspective on mental health. Psychol Bull. 1988;103(2):193–210. 3283814

[56] Weinstein N. Unrealistic optimism about future life events. J Pers Soc Psychol. 1980;39(5):806–20.

[57] Kogut T, Beyth-Marom R. Who helps more? How self-other discrepancies influence decisions in helping situations. J Judgment Decis Making. 2008;3(8):595–606.

[58] Epley N, Gilovich T. The anchoring-and-adjustment heuristic: why the adjustments are insufficient. Psychol Sci. 2006;17(4):311–8. doi: 10.1111/j.1467-9280.2006.01704.x 16623688

[59] Galizzi MM, Navarro-Martinez D. On the external validity of social preference games: a systematic lab-field study. Management Science. 2019;65(3):976–1002.

[60] Gurven M, Winking J. Collective action in action: prosocial behavior in and out of the laboratory. Am Anthropol. 2008;110(2):179–90.

[61] Winking J, Mizer N. Natural-field dictator game shows no altruistic giving. Evol Hum Behav. 2013;34(4):288–93.

[62] Dickert S, Sagara N, Slovic P. Affective motivations to help others: a two‐stage model of donation decisions. J Behav Decis Mak. 2011;24(4):361–76.

[63] Erlandsson A, Dickert S. A typology of psychological mechanisms underlying prosocial decisions. Nonprofit Volunt Sect Q. 2024;63(1):1–15. doi: 08997640241280983

[64] Shlefer S, Kogut T. How did it feel? Affect as a feedback system in repeated donation decisions. J Exp Soc Psychol. 2021;97:104203.

[65] Ajzen I, Fishbein M. Understanding attitudes and predicting social behavior. 1980.

[66] Kredentser MS, Fabrigar LR, Smith SM, Fulton K. Following what people think we should do versus what people actually do: Elaboration as a moderator of the impact of descriptive and injunctive norms. Soc Psychol Pers Sci. 2012;3(3):341–7.

[67] Schwartz SH. Are there universal aspects in the structure and contents of human values?. J Soc Issues. 1994;50(4):19–45. doi: 10.1111/j.1540-4560.1994.tb01196.x

[68] Human S, Capraro V. The effect of nudging personal and injunctive norms on the trade-off between objective equality and efficiency. 2020.

[69] Faul F, Erdfelder E, Lang A-G, Buchner A. G*Power 3: a flexible statistical power analysis program for the social, behavioral, and biomedical sciences. Behav Res Methods. 2007;39(2):175–91. doi: 10.3758/bf03193146 17695343

[70] Andreoni J. Warm-glow versus cold-prickle: the effects of positive and negative framing on cooperation in experiments. Q J Econ. 1995;110(1):1–21. doi: 10.2307/2118508

[71] Hayes AF. Introduction to mediation, moderation, and conditional process analysis: a regression-based approach. 2013.

[72] Moche H, Gordon-Hecker T, Kogut T, Västfjäll D. Thinking, good and bad? Deliberative thinking and the singularity effect in charitable giving. J Judgment Decis Making. 2022;17(1):14–30.

[73] Small D, Loewenstein G, Slovic P. Sympathy and callousness: the impact of deliberative thought on donations to identifiable and statistical victims. Organ Behav Hum Decis Process. 2007;102(1):143–53.

[74] Tversky A, Kahneman D. Judgment under uncertainty: heuristics and biases. Science. 1974;185(4157):1124–31. doi: 10.1126/science.185.4157.1124 17835457

[75] Rubaltelli E, Agnoli S. The emotional cost of charitable donations. Cogn Emot. 2012;26(5):769–85. doi: 10.1080/02699931.2011.613921 22077801

[76] Weyant J, Smith S. Getting more by asking for less: the effects of request size on donations of charity 1. J Appl Soc Psychol. 1987;17(4):392–400.

[77] Haggag K, Paci G. Default tips. Am Econ J Appl Econ. 2014;6(3):1–19.25485039

[78] Gråd E, Erlandsson A, Tinghög G. Do nudges crowd out prosocial behavior?. Behav Public Policy. 2021;8(1):107–20. doi: 10.1017/bpp.2021.10

[79] Sunstein CR. Nudges that fail. Behav Public Policy. 2017;1(1):4–25. doi: 10.1017/bpp.2016.3

[80] Brehm J, Brehm S. Psychological reactance: a theory of freedom and control. 1981.

[81] Malkoc SA. Why are donors more generous with time than money? The role of perceived control over donations on charitable giving. J Consum Res. 2022.

[82] Pavey L, Sparks P. Reactance, autonomy and paths to persuasion: Examining perceptions of threats to freedom and informational value. Motiv Emot. 2009;33(3):277–90. doi: 10.1007/s11031-009-9137-1

[83] Capraro V, Jagfeld G, Klein R, Mul M, de Pol I van. Increasing altruistic and cooperative behaviour with simple moral nudges. Sci Rep. 2019;9(1):11880. doi: 10.1038/s41598-019-48094-4 31417106 PMC6695418

[84] Schwartz SH. Normative influences on altruism. Adv Exp Soc Psychol. 1977:221–79. doi: 10.1016/s0065-2601(08)60358-5

[85] Hopper J, Nielsen J. Recycling as altruistic behavior: normative and behavioral strategies to expand participation in a community recycling program. Environ Behav. 1991;23(2):195–220.

[86] Nordlund A, Garvill J. Effects of values, problem awareness, and personal norm on willingness to reduce personal car use. J Environ Psychol. 2003;23(4):339–47.

[87] Pristl AC, Kilian S, Mann A. When does a social norm catch the worm? Disentangling social normative influences on sustainable consumption behaviour. J Consum Behav. 2021;20(3):635–54.

[88] Baumeister RF, Masicampo EJ, Vohs KD. Do conscious thoughts cause behavior?. Annu Rev Psychol. 2011;62:331–61. doi: 10.1146/annurev.psych.093008.131126 21126180

[89] Pittarello A, Kogut T. To ask or not to ask: enhancing donations to nonprofits by Soliciting opinions upfront, rather than donations. J Bus Psychol. 2022;37:759–73.

