# Supplementary material for: Thinking of norms—or being told what they are: The effect of social information on donation decisions
Source: PLoS One. 2025 Apr 25;20(4):e0321547. doi: 10.1371/journal.pone.0321547 (PMC12027029; doi:10.1371/journal.pone.0321547)
Supplement: S1 File — . (DOCX) [file pone.0321547.s001.docx]

**Thinking of Norms—or Being Told What They Are:**

**The Effect of Social Information on Donation Decisions**

**Supplementary Materials**

The following are the additional measured variables that were assessed in an exploratory fashion at the end of each experiment, and additional analyses.

**Additional Measured Variables**

In both studies, following their emotions' ratings, participants were asked two exploratory questions (rated on the same 1–7 scale):

1. How difficult was it for you to make your decision?
2. In your opinion, how significant is a donation for the organization?

Finally, they were asked “*How much (between NIS 0–100) would you recommend to the average student taking part in the study to donate, if they were to win the raffle*?” and answered demographics questions.

Study 1- Additional Analyses

Recommended Behavior

To examine the effect of Norm Type and Anchor on the recommended behavior (i.e., the amount the participants recommended to others to donate), a two-way ANOVA of Norm Type and Anchor on the recommended amount was conducted. The results revealed a significant main effect for Anchor: *F*(1, 243) = 14.49, *p* < 0.001, *η_p_^2^* = .056—such that in the high-anchor conditions, the recommended amount was higher (*M* = 50.73, *SD* = 26.50) than in the low-anchor ones (*M* = 37.62, *SD* = 27.37) .The interaction between Anchor and Norm Type was also significant, *F*(2, 243) = 4.49, *p* = 0.012, *η_p_^2^* = .036, and is presented in Fig. 1. A simple-effect analysis suggests that the effect of the anchor was significant in the Injunctive Norm, *F*(1, 243) = 10.12, *p* = .002, *η_p_^2^* = .040, and in the Descriptive Norm, *F*(1, 243) = 12.73 , *p* < .001, *η_p_^2^* = .050—such that with a high anchor, participants recommended a higher amount (Injunctive Norm: *M* = 53.87, *SD* = 22.05; Descriptive Norm: *M* = 53.05, *SD* = 28.26) than with a low anchor (Injunctive Norm: *M* = 34.61, *SD* = 23.92 ; Descriptive Norm: *M* = 31.77, *SD* = 22.96)—but not in the control *F*(1, 243) = 0.049, *p* = .825, *η_p_^2^* = .000.


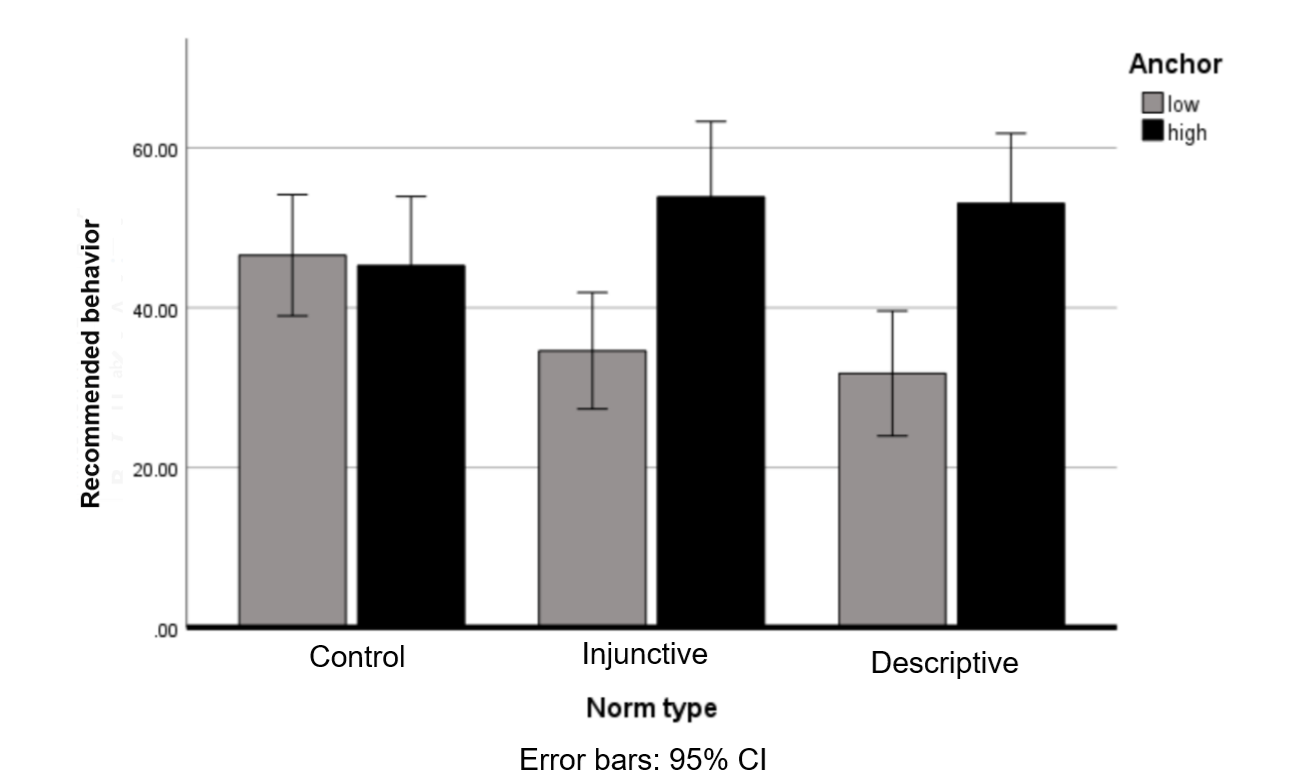


Fig. 1. Recommended behavior, as a function of Norm Type and Anchor

Study 2: Additional Analyses

Donation Amount

Table 1. Means, Standard Deviations and the results of the One-way ANOVA on donation amount reported in the main text:

| **Condition** | **Descriptive**  **M SD** | **Injunctive**  **M SD** | **Control**  **M SD** | **F (2, 389)** | ***p*** | ***η_p_^2^*** |
| --- | --- | --- | --- | --- | --- | --- |
| **Norm Type** | 31.64 32.19 45.65 38.79 37.02 34.24 | | | 5.135 | *.006* | *.026* |

The same analysis described in the main manuscript, on donation amount as a function of norm-type, with donors only, revealed a significant effect for the norm type, F (2, 281) = 3.59, p = .029, *η_p_^2^* = .025

Recommended Behavior

To examine the effect of Norm Type on the recommended behavior (i.e. the donation amount that participants recommended to the average student taking part in the study), I conducted a one-way ANOVA on the recommended amount, with Norm Type as a factor. The results revealed a significant effect for Norm Type, *F*(2, 379) = 3.05, *p* = .048, *η_p_^2^* = .016)—as shown in Fig.1. A post-hoc analysis further suggested that participants in the Injunctive Norm condition recommended significantly higher amounts (*M* = 43.65, *SD* = 29.88) than those in the Descriptive Norm condition (*M* = 35.10, *SD* = 27.29), *p* = 0.05. The comparisons between the two norms and the control condition were not significant (control-descriptive, *p* = .393; control-injunctive, *p* =.104).


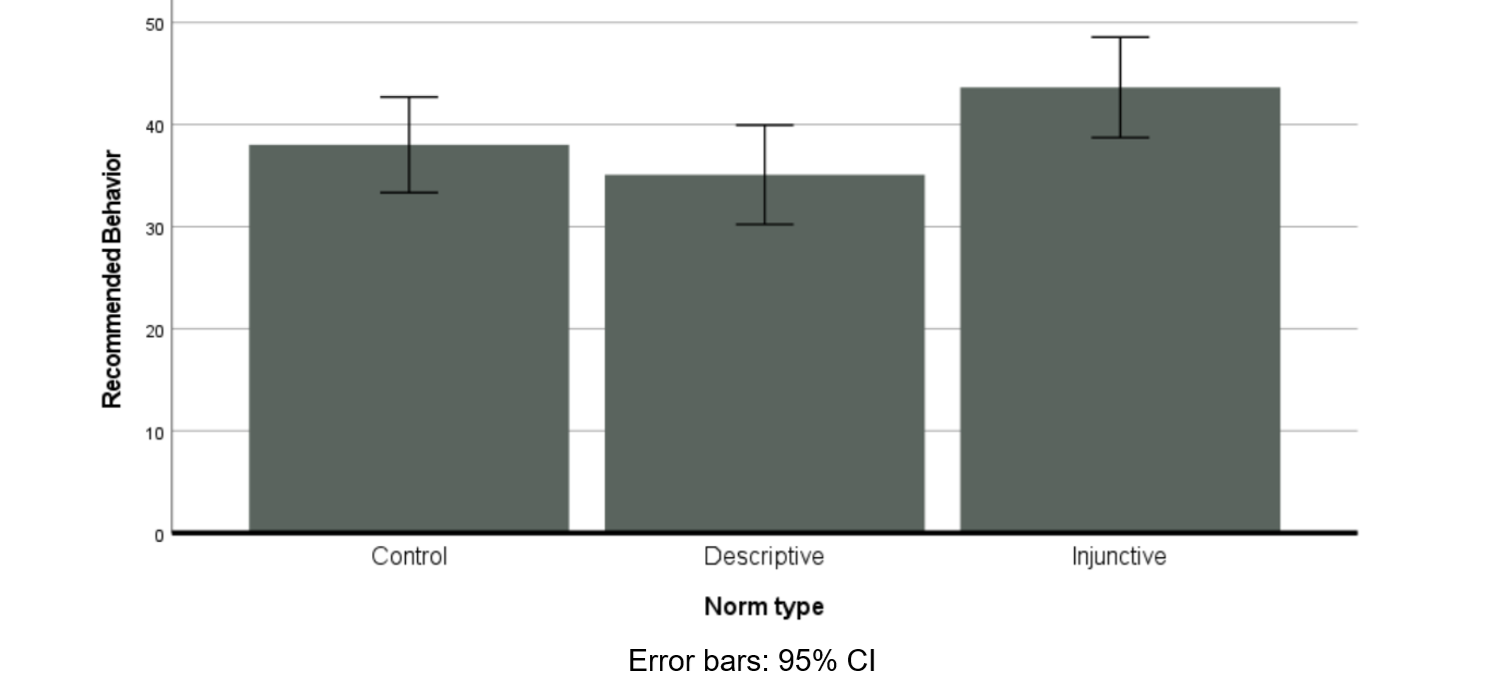


Fig. 1: Recommended behavior (the amount recommended to others to donate) as a function of Norm Type.
